# A New Method of Hardening Rubber

**Published:** 1872-05

**Authors:** James E. McCabe

**Affiliations:** Bolivar, Mo.


### ARTICLE XIII.

### *A New Method of Hardening Rubber.*

By James E. McCabe, Bolivar, Mo.

The plate to be hardened or vulcanized, is flasked in the usual manner, the same apparatus being used as for steam. The difference is simply this:

I use ammonical gas instead of the steam. The gas is formed of equal parts of chloride of ammonia and hydrate of lime—common lime slacked in a covered vessel. For a single case vulcanizer, 15 or 20 grains of each ingredient, finely pulverized, should be placed in a tin box, sufficiently perforated with small holes to allow the gas to escape freely, which will generate very rapidly from about 60°. Place the box in the vulcanizer with the flask and apply the heat in the usual manner, allowing about half an hour for the mercury to rise to 320° so that the gas may have time to penetrate the plaster, then vulcanize for fifteen or twenty minutes time. The above time will harden the same rubber that requires by the old process an hour and a half, and I believe, leaving it in a much better condition for the mouth. The chlorine of ammonia being set free, becomes a powerful disinfectant, removing the bad effects of the sulphur and coloring matter of the rubber, and I find without injury to it.

The gas can be formed by other means than herein stated but I believe this to be the best method, and taking time into consideration, is cheaper than water. It costs but a trifle to procure the ingredients used, and in large establishments for hardening rubber goods, the gas can be converted into spirits of ammonia with a profit. I intend to introduce it as a medium for hardening rubber for all purposes. In regard to the vulcanizer, one made of iron, costing $2.50 would be better than the copper ones now in use from the fact that gas acts on the copper, but this can be prevented by oiling the vulcanizer before using, and cleaning afterwards. It is well known that ammonical gas is not explosive nor inflammable; in this respect it is safer than steam.

The flasks should be perforated, top and bottom, and in fact all around to allow the gas to penetrate the plaster. For a close flask, about thirty grains each of the ammonia and lime should be used, producing a greater volume of gas and consequently more condensed and more penetrating. Ammonia preserves iron, and consequently that would be considerable of an item to dentists, and, in fact, to all who harden rubber.—*Missouri Dental Journal.*

